# The Association Between Measures of Prepregnancy Insulin Resistance and Sensitivity with Subsequent Risk of Gestational Diabetes

**DOI:** 10.1210/jendso/bvaf227

**Published:** 2026-01-12

**Authors:** Shalmali Bane, Fei Xu, Assiamira Ferrara, Sneha Sridhar, Charles P Quesenberry, Monique M Hedderson

**Affiliations:** Division of Research, Kaiser Permanente Northern California, Pleasanton, CA 94588, USA; Center for Upstream Prevention of Adiposity and Diabetes Mellitus (UPSTREAM), Division of Research, Kaiser Permanente Northern California, Pleasanton, CA 94588, USA; Division of Research, Kaiser Permanente Northern California, Pleasanton, CA 94588, USA; Division of Research, Kaiser Permanente Northern California, Pleasanton, CA 94588, USA; Center for Upstream Prevention of Adiposity and Diabetes Mellitus (UPSTREAM), Division of Research, Kaiser Permanente Northern California, Pleasanton, CA 94588, USA; Division of Research, Kaiser Permanente Northern California, Pleasanton, CA 94588, USA; Division of Research, Kaiser Permanente Northern California, Pleasanton, CA 94588, USA; Division of Research, Kaiser Permanente Northern California, Pleasanton, CA 94588, USA; Center for Upstream Prevention of Adiposity and Diabetes Mellitus (UPSTREAM), Division of Research, Kaiser Permanente Northern California, Pleasanton, CA 94588, USA

**Keywords:** gestational diabetes, HOMA-IR, race and ethnicity, diabetes in pregnancy, insulin resistance

## Abstract

**Context:**

Little is known about the association between prepregnancy homeostasis model assessment of insulin resistance (HOMA-IR) and homeostasis model assessment of β-cell function (HOMA-β) levels and risk of gestational diabetes mellitus (GDM).

**Objective:**

To examine the association between prepregnancy levels of HOMA-IR and HOMA-β and risk of GDM and whether these associations vary by race and ethnicity.

**Research Design and Methods:**

We conducted a nested case-control study among women who had a serum sample from 1984 to 1996 used to measure glucose and insulin and a subsequent pregnancy from 1984 to 2009. GDM cases (n = 254) were matched with 2 randomly selected control subjects (n = 497) on serum collection date, age at collection, number of intervening pregnancies, and age at delivery. We used conditional logistic regression to estimate odds ratios (ORs) and 95% confidence intervals (CIs) for GDM by HOMA-IR and HOMA- β, overall and stratified by race and ethnicity.

**Results:**

Increasing levels of HOMA-IR were associated with higher odds of GDM (adjusted OR for 1 SD increase: 1.31 [95% CI 1.12,1.54]) and tertile 3 compared to tertile 1 (3.04 [95% CI 1.81, 5.12]). Models stratified by race/ethnicity suggest this association was significant among Asian and non-Hispanic Black populations for tertile 3 (3.83 [95% CI 1.39, 10.57] and 2.18 [95% CI 1.03, 4.61], respectively). Among non-Hispanic Black individuals, a 1 SD increase in HOMA-IR also increased risk of GDM (OR: 1.59, 95% CI 1.17, 2.17). There was no significant association between HOMA-IR and GDM among non-Hispanic White or Hispanic individuals. There was no association between HOMA-β and GDM risk.

**Conclusion:**

Higher HOMA-IR levels before pregnancy were associated with an elevated risk of GDM.

Gestational diabetes mellitus (GDM), affecting upwards of 8% of pregnant individuals, is characterized by glucose intolerance with onset first recognized during pregnancy [1]. Gestational diabetes is associated with increasing risk of gestational hypertension, preeclampsia, and cesarean birth during the perinatal period [2]; subsequent maternal risk of cardiovascular disease; and type 2 diabetes (T2D) [2, 3], and infant risk of developing obesity or T2D [4]. Pregnancy induces increased maternal insulin resistance, resulting in increasing insulin production by pancreatic β-cells [5]. In pregnancies complicated by GDM, the pancreatic β-cells are unable to compensate for this insulin resistance, resulting in impaired glucose tolerance, or GDM, similar to the pathophysiology of T2D [5, 6].

Almost half of individuals identified by universal screening methods for GDM lack any clinically available established risk factors such as older age, obesity, and family history [7, 8]. Thus, biomarkers represent a promising avenue for the early prediction of GDM risk and identification of individuals among whom interventions for GDM prevention can be targeted [9, 10]. Higher-levels of insulin resistance, measured by the homeostasis model assessment of insulin resistance (HOMA-IR) and impaired insulin secretion, measured by the homeostasis model assessment of β-cell function (HOMA-β) when assessed during pregnancy are associated with GDM risk [11-13]. However, very little is known about the association between prepregnancy HOMA-IR or HOMA-β levels and risk of GDM [14, 15]. Given that the measurement of glucose and insulin is easy and relatively inexpensive, their measurement in reproductive-aged women could help identify patients at high risk of GDM to target for preconception prevention strategies.

The risk of GDM varies widely by race and ethnicity, with Asian, American Indian, and Hispanic individuals from the United States experiencing elevated risk, relative to their non-Hispanic White or Black counterparts [16-18]. However, it is important to note that the roles of biological factors are likely complemented by environmental and structural determinants that contribute to racial and ethnic disparities in GDM [19-21].

Understanding the relationship of prepregnancy insulin resistance and secretion with GDM can help us better understand the etiology of GDM and whether it varies by race and ethnicity. This could also help identify individuals at risk for GDM as well as inform risk stratification and the development of tailored preconception interventions. Using a nested case-control cohort from a study of women who took part in a comprehensive health examination from 1984 to 1996 and had a subsequent pregnancy at Kaiser Permanente Northern California (KPNC), we aimed to examine whether serum levels of HOMA-IR and HOMA-β levels measured on average 7 years before pregnancy were independently associated with risk of GDM and to assess whether these possible associations varied by race and ethnicity and prepregnancy body mass index (BMI).

## Materials and Methods

### Setting

This study was conducted at KPNC, an integrated healthcare delivery system that provides medical care for over 4.6 million members, representing 30% of the service area (which spans 14 counties of the Greater Bay Area and the California Central Valley from Sacramento to Fresno). KPNC membership is representative of the demographic characteristics of the entire population from this geographic area [22-24].

Between 1984 and 1996, female KPNC members could complete a voluntary and comprehensive health examination [the multiphasic health checkup (MHC)] at the Kaiser Permanente Oakland Medical Center. The MHC included a clinic visit for the completion of questionnaires and clinical measurements, including a random blood draw with an extra serum sample that was collected and stored at −40 °C for future use [25]. Among women 15 to 45 years who participated in the MHC from 1984 to 1996 (n = 27 743 with clinical data, questionnaire data, and an extra serum sample), we identified 4098 women who subsequently delivered an infant by 2010 through the KPNC hospitalization database and the Pregnancy Glucose Tolerance and GDM Registry, an active surveillance registry that annually identifies all pregnancies resulting in a livebirth or stillbirth among KPNC members [26]. We identified and excluded women with prepregnancy diabetes using clinical screening data from the GDM Registry [27]. The registry also captures the results of all screening and diagnostic tests for GDM from the KPNC electronic health record (EHR) laboratory database (from 1994 onward).

### Study Design

**Figure 1.**
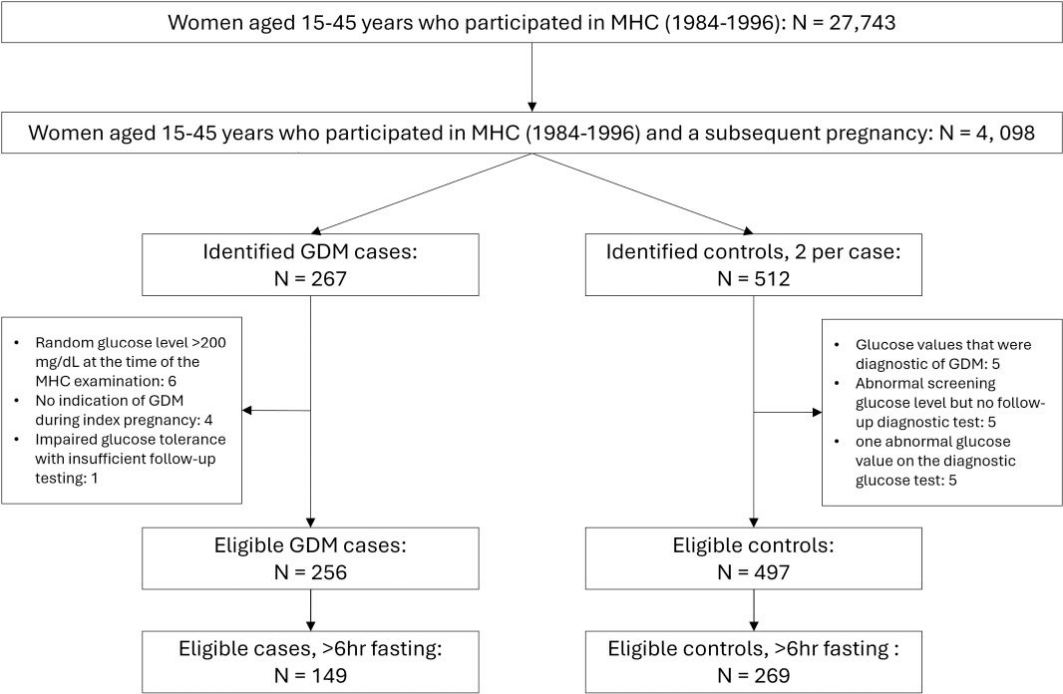
Case control study flow chart. Abbreviations: GDM, gestational diabetes mellitus; MHC, multiphasic checkup.

We conducted a nested case-control study within a cohort of 4098 women who took part in an MHC examination, had an extra tube of serum stored for future use, and had a subsequent pregnancy at KPNC (on average, 7 years after MHC examination; Fig. 1). Case patients were individuals who developed GDM, and 2 control subjects were selected per case among women who did not meet the definition of GDM among cases.

### Case Definition

Using the KPNC EHR, 267 cases were identified as either having (1) glucose values obtained during a standard 100-g, 3-hour oral glucose tolerance test that met the Carpenter and Coustan plasma glucose thresholds for GDM [28] (n = 228) or (2) a hospital discharge diagnosis of GDM in the electronic hospital discharge database for pregnancies occurring before the EHR laboratory data were available, prior to 1994 (n = 39); both methods were confirmed via standardized medical chart review conducted by trained abstractors. The following plasma glucose thresholds were considered as GDM cases: fasting, 5.3 mmol/L [95 mg/dL]; 1-hour, 10.0 mmol/L [180 mg/dL]; 2-hour, 8.6 mmol/L [155 mg/dL]; 3-hour, 7.8 mmol/L [140 mg/dL]. Cases were excluded if they had a random glucose level >200 mg/dL at the time of the MHC examination (n = 6), no indication of GDM during the index pregnancy (n = 4), or impaired glucose tolerance with insufficient follow-up testing (n = 1). Thus, the eligible number of confirmed cases was 256. We selected the first diagnosis of GDM after the MHC examination.

### Controls

Control subjects were randomly selected among women without an indication of GDM. Two controls were individually matched to each case based on (1) the year of MHC serum collection date (±3 months), (2) age at MHC serum collection (±2 years), and (3) age at delivery of the index pregnancy (±2 years). We matched for the year of serum collection to ensure that any potential degradation in the quality of the serum over time was comparable between cases and controls. Since the prevalence of GDM is higher among older patients, we matched on age at serum collection and age at delivery. Lastly, we matched on the number of pregnancies to account for any differences in pregnancies between the initial examination and the index pregnancy. We excluded control subjects if they had glucose values diagnostic of GDM found during medical chart abstraction (n = 5), had an abnormal screening glucose level but no follow-up diagnostic glucose test (n = 5), or had 1 abnormal glucose value on the diagnostic glucose test (n = 5), suggestive of “mild” GDM. Of the 512 matched control subjects identified, 497 were eligible.

### Exposure

Insulin resistance, based on HOMA-IR, was calculated as: glucose (mmol/L)×fasting insulin (μU/mL)/22.5, where glucose was measured in millimoles per liter and insulin in milliunits per milliliter; this surrogate measure has been validated against the gold standard and is used widely in clinical and epidemiological studies [29-31]. Insulin sensitivity or β-cell function (HOMA-β) was calculated as: 20 × fasting insulin (μU/mL)/fasting glucose (mmol/L) – 3.5 [32]. For measuring insulin, serum samples from the MHC were thawed, aliquoted, and transported in batches on dry ice to the laboratory of Dr. Peter Havel at the University of California, Davis. Serum glucose was measured using the hexokinase method by the regional laboratory of KPNC at the time of the MHC exam. Both laboratories participate in the College of American Pathologists' accreditation and monitoring program.

### Covariates

Information on age, race and ethnicity, education level, alcohol consumption, and fasting status (≥6 hours since last food ingestion) was collected with a self-administered questionnaire at the MHC examination [25]. To calculate weight change (kilograms per year) from the MHC examination to the start of pregnancy, prepregnancy weight was abstracted from the medical record as a measured weight in the first trimester of pregnancy or as self-reported prepregnancy weight if a measured weight was unavailable. Baseline BMI was calculated with standard methods from height that was measured with a stadiometer and weight using a balance beam scale at the time of MHC.

### Statistical Analysis

We used conditional logistic regression (with a binomial distribution and a logit link) to estimate odds ratios (ORs) and 95% confidence intervals (CIs) for the association of GDM with HOMA-IR and HOMA-β, both as a continuous measure and in tertiles (as defined among control subjects, reference group = lowest tertile), to aid in interpretability while optimizing statistical power. In addition to variables used in case-control matching, estimates were adjusted as follows: model 0: adjusted for fasting status; model 1: further adjusted for family history of diabetes, alcohol use, race and ethnicity (all from the time of the MHC examination), and parity; and model 2: further adjusted for baseline BMI (continuous) from the time of the MHC examination. Next, we assessed the role of race/ethnicity as well as baseline BMI (<25 vs ≥ 25), which we a priori hypothesized as biologically plausible effect modifiers of the relationship of HOMA-IR and HOMA-β with GDM. To do so, we stratified models by hypothesized effect modifiers, using the same cutoffs for the continuous measures and tertiles from the overall sample and the lowest quartile of HOMA-IR (ie, lowest HOMA-IR levels) or HOMA-β (ie, highest HOMA-β levels) as the reference group; these were unconditional logistic regression models with model 0 adjusted for matching variables (MHC serum collection year, difference between age at MHC serum collection and age at delivery, and age at delivery). Additionally, we formally tested for effect modification by adding a cross-product (interaction) term for hypothesized effect modifier and HOMA-IR or HOMA-β to the model; for race and ethnicity, we considered non-Hispanic White race/ethnicity as the reference group. We considered a lenient *P* < .1 as evidence of effect modification, as this analysis was powered for the main effects and not interaction effects.

As a sensitivity analysis, we repeated the analysis among a restricted sample of individuals who reported 6 or more hours of fasting prior to serum collection (n = 418). We ran unconditional logistic regression models among this sample, with model 0 adjusted for matching variables (MHC serum collection year, difference between age at MHC serum collection and age at delivery, and age at delivery). This study was approved by the human subjects committee of the Kaiser Foundation Research Institute, and the analysis was conducted using SAS 9.4.

## Results

In the overall sample of 751 individuals, 254 were GDM cases and 497 were matched controls. On average, GDM cases had a higher prevalence of prepregnancy obesity (22.8% vs 10.3% for BMI ≥ 30), lower educational attainment (36.2% vs 43.1% for ≥16 years), higher prevalence of family history of diabetes (58.7% vs 38.6%), and less alcohol use (29.1% vs 16.3% for no use) and were more often Asian (31.1% vs 16.9%) or Hispanic (13.8% vs 8.7%) and less often non-Hispanic White (19.7% vs 37.4%) or non-Hispanic Black (35.4% vs 37.0%), compared to controls. The MHC health examination was performed, on average, 6.7 and 6.0 years before conception for cases and control subjects, respectively (Table 1). HOMA-IR and HOMA-β levels were higher among GDM cases compared with controls: 3.6 vs 2.5 and 261.8 vs 228, respectively. Comparisons between the cases and controls were similar for the sample of patients with a blood draw after ≥6 hours of fasting; their HOMA-IR and HOMA-β levels were slightly lower than the overall population (3.1 vs 2.3 and 233.4 vs 201.6 among cases and controls, respectively).

**Table 1.** Characteristics of GDM cases and controls, overall sample (n = 751) and among patients with a fasting blood draw (n = 418)

Characteristic	Overall sample (n = 751)		Patients with a fasting blood draw (n = 418)	
	GDM cases	Controls	GDM cases	Controls
	(n = 254)	(n = 497)	(n = 149)	(n = 269)
Age at index delivery (years), n (%)				
< 30	39 (15.4)	80 (16.1)	21 (14.1)	36 (13.4)
30-<35	73 (28.7)	145 (29.2)	45 (30.2)	77 (28.6)
35-<40	101 (39.8)	183 (36.8)	62 (41.6)	103 (38.3)
≥ 40	41 (16.1)	89 (17.9)	21 (14.1)	53 (19.7)
BMI (kg/m^2^), n (%)				
< 18.5	17 (6.7)	28 (5.6)	11 (7.4)	10 (3.7)
18.5-<25.0	115 (45.3)	325 (65.4)	70 (47.0)	187 (69.5)
25.0-<30.0	64 (25.2)	93 (18.7)	36 (24.2)	55 (20.4)
≥ 30	58 (22.8)	51 (10.3)	32 (21.5)	17 (6.3)
Education, years (IQR)				
≤ 12	73 (28.7)	119 (23.9)	46 (30.9)	63 (23.4)
13-<16	84 (33.1)	157 (31.6)	45 (30.2)	84 (31.2)
≥ 16	92 (36.2)	214 (43.1)	56 (37.6)	121 (45.0)
Missing	5 (2.0)	7 (1.4)	2 (1.3)	1 (0.4)
Rate of gestational weight gain (kg/week)*^a^*				
Parity, n (%)				
0	140 (55.1)	278 (55.9)	75 (50.3)	158 (58.7)
1	47 (18.5)	106 (21.3)	26 (17.4)	55 (20.4)
≥2	44 (17.3)	70 (14.1)	30 (20.1)	35 (13.0)
Missing	23 (9.1)	43 (8.7)	18 (12.1)	21 (7.8)
Family history of diabetes	149 (58.7)	192 (38.6)	89 (59.7)	96 (35.7)
Alcohol use, n (%)				
None	74 (29.1)	81 (16.3)	42 (28.2)	36 (13.4)
Any use	147 (57.9)	346 (69.6)	80 (53.7)	195 (72.5)
Missing	33 (13.0)	70 (14.1)	27 (18.1)	38 (14.1)
Race and ethnicity, n (%)				
Asian	79 (31.1)	84 (16.9)	49 (32.9)	40 (14.9)
Non-Hispanic Black	90 (35.4)	184 (37.0)	48 (32.2)	95 (35.3)
Hispanic	35 (13.8)	43 (8.7)	19 (12.8)	23 (8.6)
Non-Hispanic White	50 (19.7)	186 (37.4)	33 (22.1)	111 (41.3)
HOMA-IR index tertile, n (%)				
T1	47 (18.5)	164 (33.0)	24 (16.1)	88 (32.7)
T2	67 (26.4)	168 (33.8)	38 (25.5)	92 (34.2)
T3	140 (55.1)	165 (33.2)	87 (58.4)	89 (33.1)
HOMA-β index tertile, n (%)				
T1	66 (26.0)	163 (32.9)	43 (28.9)	88 (32.8)
T2	91 (35.8)	169 (34.1)	48 (32.2)	91 (34.0)
T3	97 (38.2)	164 (33.1)	58 (38.9)	89 (33.2)
Age at MHC exam (years), median (IQR)	27.8 (23.9-32.5)	27.7 (24.3-31.9)	28.3 (24.2-32.6)	27.9 (24.3-31.8)
Time between MHC exam and delivery (years), median (IQR)	6.7 (3.7-10.1)	6.0 (3.3-9.7)	6.8 (3.8-9.2)	6.6 (3.8-10.4)
HOMA-IR index, median (IQR)	3.6 (2.4-6.2)	2.5 (1.8-3.9)	3.1 (2.1-4.7)	2.3 (1.7-3.3)
HOMA-β index, median (IQR)	261.8 (168.5-414.5)	228.6 (154.6-402.4)	233.4 (148.3-339.2)	201.6 (140.9-320.3)

Abbreviations: BMI, body mass index; GDM, gestational diabetes mellitus; HOMA-β, homeostasis model assessment of β-cell function; HOMA-IR, homeostasis model assessment of insulin resistance; IQR, interquartile range; MHC, multiphasic checkup.

^a^Weight change in kilograms per week from beginning of index pregnancy until screening glucose (measurement obtained 1 hour after the 50-g oral challenge). Data were unavailable for 21 cases and 51 control subjects in the overall cohort and 11 cases and 23 controls in the sample of patients with fasting blood draws.

Increasing levels of HOMA-IR were associated with higher odds of GDM (unadjusted OR for 1 SD: 1.48; 95% CI 1.26-1.76; Table 2). Adjustment led to a slight attenuation of the estimates (fully adjusted OR: 1.31; 95% CI 1.12-1.54). For tertiles of HOMA-IR, tertile 3 (range: 3.3-60.0, median: 5.4) as compared with tertile 1 (range: 0.2-1.9, median: 1.5) was associated with a significantly higher odds of GDM (fully adjusted OR: 3.04; 95% CI 1.81-5.12); estimates for tertile 2 (range: 2.0-3.2, median: 2.6) were >1 but not statistically significant (1.28; 95% CI 0.79-2.08). In a sensitivity analysis among women who had the blood draw after ≥6 hours of fasting, the unadjusted and adjusted estimates for continuous HOMA-IR were 1.42 (95% CI 1.14-1.77) and 1.23 (95% CI 0.99-1.51), respectively; the fully adjusted estimate for tertile 3, relative to tertile 1, was lower than the full sample but still elevated and statistically significant: 2.49 (95% CI 1.33-4.65) (Table A.1). Adjusted estimates for HOMA-β levels were not associated with increased risk of GDM in the full sample or sample of patients with a blood draw after ≥6 hours of fasting (Table 2 and A.1).

**Table 2.** Sequentially adjusted ORs (95% CI) for the association between HOMA-IR and HOMA-β with GDM, overall sample (n = 751)*^a^*

Independent variables	Range	OR (95% CI)		
		Model 0*^b^*	Model 1*^c^*	Model 2*^d^*
HOMA-IR				
Continuous measure*^e^*	—	1.48 (1.26-1.76)	1.42(1.20-1.67)	1.31 (1.12-1.54)
Tertiles				
T1	0.2-1.9		(reference)	
T2	2.0-3.2	1.42 (0.93-2.17)	1.42 (0.89-2.27)	1.28 (0.79-2.08)
T3	3.3-60.0	3.79 (2.44-5.89)	4.05 (2.47-6.64)	3.04 (1.81-5.12)
HOMA-β				
Continuous measure *^f^*	—	1.11 (0.94-1.30)	1.14 (0.96-1.36)	1.05 (0.87-1.27)
Tertiles				
T1	−2710.1-174.6		(reference)	
T2	174.7-320.8	1.46 (0.97-2.19)	1.44 (0.93-2.24)	1.27 (0.80-2.03)
T3	320.9-3632.5	1.67 (1.09-2.54)	1.64 (1.03-2.63)	1.20 (0.72-1.98)

Abbreviations: BMI, body mass index; CI, confidence interval; GDM, gestational diabetes mellitus; HOMA-β, homeostasis model assessment of β-cell function; HOMA-IR, homeostasis model assessment of insulin resistance; OR, odds ratio.

^a^Model is a conditional logistic regression.

^b^Model 0 is adjusted for fasting status.

^c^Model 1 is model 0 with additional adjustment for race and ethnicity, family history, alcohol use, and parity.

^d^Model 2 is model 1 with additional adjustment for BMI.

^e^OR is interpreted as increased in odds for every 1 SD difference (4.2).

^f^OR is interpreted as increased in odds for every 1 SD difference (455.3).

In stratified models, the unadjusted estimates for continuous HOMA-IR for all racial and ethnic groups were associated with increased odds of GDM: 1.58 (95% CI 1.06-2.35) for Asian individuals, 1.77 (95% CI 1.30-2.41) for non-Hispanic Black individuals, 3.25 (95% CI 1.38-7.69) for Hispanic individuals and 1.37 (95% CI 1.00-1.88) for non-Hispanic White individuals (Table 3). Similarly, the unadjusted associations between the tertile 3 of HOMA-IR, relative to tertile 1, were significant among all racial and ethnic groups. However, in the fully adjusted models, no associations remained among non-Hispanic White or Hispanic individuals; sample size among Hispanic individuals was small (n = 78) and, as such, this analysis could be underpowered. The formal tests of the heterogeneity of effects across race and ethnicity were largely not significant for HOMA-β and HOMA-IR (Table A.2). When stratifying the full sample by baseline BMI, estimates were similar to the main analysis; ie, the associations for continuous HOMA-IR (1.44, 95% CI 1.12-1.85 and 1.29, 95% CI 1.05-1.57, for BMI <25 and BMI ≥25, respectively) and tertile 3 of HOMA-IR (2.49, 95% CI 1.41-4.41 and 3.31; 95% CI 1.42-7.74, for BMI <25 and BMI ≥25, respectively) were elevated and significant. (Table A.4). The formal tests of the heterogeneity of effects by baseline BMI were largely not significant for any examined associations (Table A.2).

**Table 3.** Sequentially adjusted ORs (95% CI) for the association between HOMA-IR and HOMA-β with GDM, overall sample (n = 751), stratified by race and ethnicity*^a^*

Independent variables	n, HOMA-IR	n, HOMA-β	HOMA-IR, OR (95% CI)			HOMA-β, OR (95% CI)		
			Model 0*^b^*	Model 1*^c^*	Model 2*^d^*	Model 0*^b^*	Model 1*^c^*	Model 2*^d^*
Asian individuals (n = 163)								
Continuous*^e^*			1.58 (1.06-2.35)	1.51 (1.00-2.30)	1.48 (0.98-2.24)	1.11 (0.64-1.91)	1.06 (0.61-1.85)	0.97 (0.55-1.74)
Tertiles								
T1	38	53		(reference)			(reference)	
T2	55	64	2.20 (0.87-5.57)	2.33 (0.88-6.16)	2.27 (0.85-6.06)	0.87 (0.40-1.90)	0.82 (0.36-1.87)	0.78 (0.34-1.78)
T3	70	46	3.96 (1.54-10.20)	4.03 (1.52-10.70)	3.83 (1.39-10.57)	1.80 (0.73-4.43)	1.76 (0.70-4.43)	1.58 (0.61-4.09)
Non-Hispanic Black individuals (n = 274)								
Continuous*^e^*			1.77 (1.30-2.41)	1.76 (1.29-2.40)	1.59 (1.17-2.17)	1.15 (0.92-1.43)	1.17 (0.93-1.48)	1.09 (0.86-1.40)
Tertile								
T1	68	52		(reference)			(reference)	
T2	76	92	0.83 (0.37-1.87)	0.76 (0.33-1.76)	0.71 (0.30-1.65)	1.25 (0.58-2.67)	1.43 (0.65-3.12)	1.22 (0.54-2.73)
T3	130	129	2.99 (1.50-5.96)	2.98 (1.47-6.01)	2.18 (1.03-4.61)	1.34 (0.65-2.79)	1.49 (0.70-3.17)	1.05 (0.47-2.35)
Hispanic individuals (n = 78)								
Continuous*^e^*			3.25 (1.38-7.69)	3.15 (1.23-8.04)	2.08 (0.85-5.10)	2.77 (0.83-9.31)	2.21 (0.59-8.25)	1.08 (0.18-6.42)
Tertile								
T1	21	20		(reference)				
T2	21	30	1.74 (0.37-8.24)	1.30 (0.21-8.09)	0.65 (0.08-5.01)	2.15 (0.54-8.60)	2.79 (0.62-12.57)	2.46 (0.38-15.75)
T3	36	28	5.16 (1.16-23.00)	5.42 (1.01-29.13)	0.92 (0.11-7.51)	3.77 (0.85-16.63)	3.43 (0.69-17.01)	0.72 (0.10-5.35)
Non-Hispanic White individuals (n = 236)								
Continuous*^e^*			1.37 (1.00-1.88)	1.35 (0.96-1.90)	1.27 (0.89-1.82)	1.05 (0.72-1.53)	1.06 (0.70-1.62)	0.95 (0.59-1.54)
Tertile								
T1	84	104		(reference)			(reference)	
T2	83	74	1.59 (0.69-3.69)	1.58 (0.65-3.84)	1.46 (0.59-3.63)	1.51 (0.71-3.25)	1.62 (0.71-3.66)	1.39 (0.59-3.27)
T3	69	58	2.74 (1.15-6.56)	2.90 (1.14-7.39)	1.92 (0.70-5.27)	1.07 (0.42-2.71)	1.16 (0.43-3.12)	0.76 (0.26-2.24)

Abbreviations: BMI, body mass index; CI, confidence interval; GDM, gestational diabetes mellitus; HOMA-β, homeostasis model assessment of β-cell function; HOMA-IR, homeostasis model assessment of insulin resistance; MHC, multiphasic checkup; OR, odds ratio.

^a^Model is an unconditional logistic regression.

^b^Model 0 is adjusted for matching variables: MHC serum collection year, age at delivery of the index pregnancy, and difference between age at MHC serum collection and age at delivery of index pregnancy as well as fasting status.

^c^Model 1 is model 0 + adjustment for race and ethnicity, family history, alcohol use, and parity.

^d^Model 2 is model 1 + adjustment for BMI.

^e^OR for 1 SD difference based on overall sample controls.

Among Asian individuals, tertile 3 of HOMA-IR was associated with higher odds of GDM (3.83; 95% CI 1.39-10.57). Among the non-Hispanic Black individuals, both tertile 3 of HOMA-IR and a 1 SD increase in levels of HOMA-IR were associated with higher odds of GDM (2.18; 95% CI 1.03-4.61 and 1.59; 95% CI 1.17-2.17, respectively). There were no significant associations in the stratified analyses for HOMA-β by race and ethnicity (Table 3).

## Discussion

In a case-control study, we found that increasing levels of prepregnancy HOMA-IR, assessed on average 6 years prior to pregnancy, were associated with an increased risk of GDM, adjusting for established GDM risk factors. This was observed for both a 1 SD increase in HOMA-IR and for the highest tertile of HOMA-IR compared with the lowest. Our findings suggest that the strength of association between HOMA-IR and GDM may differ by racial and ethnic subgroups. There was no significant association between HOMA-IR and odds of GDM among non-Hispanic White or Hispanic individuals, whereas the association between higher HOMA-IR and elevated risk of GDM was present among Asian and non-Hispanic Black individuals in adjusted models. There were no observed associations between prepregnancy HOMA-β levels with risk of GDM.

These findings are in line with prior work showing the association between HOMA-IR measured during pregnancy and subsequent odds of GDM. A recent longitudinal study from Iran found that the highest tertile of HOMA-IR in early pregnancy was associated with increased odds of GDM (OR: 3.2; 95% CI 1.6-6.2), while the signal was not as strong for the second tertile (OR: 1.6; 95% CI 0.9-3.1) [33]. In addition, an observational cohort study of 700 pregnant women from China found that HOMA-IR measured in early pregnancy was associated with increased odds of GDM (1.37; 95% CI 1.13-1.67) [11]. Results from another study from China also demonstrated that the odds of GDM varied by first-trimester BMI, with the odds being highest among those with greatest BMI (OR 2.94; 95% CI 2.15-4.02 among first-trimester BMI < 24 kg/m^2^ compared with 9.42; 95% CI 1.71-51.77 among first-trimester BMI ≥ 28.0 kg/m^2^) [12]. Our results stratified by baseline BMI also showed the magnitude of the effect estimate between the highest tertile of HOMA-IR and GDM, compared to the lowest tertile, was slightly elevated among those with higher BMI. Contrary to our hypothesis, tests for interaction by baseline BMI were not significant in our study, which may have been due to a lack of power; we explored stratified analyses to assess if there was suggestion that the magnitude of effects varied by hypothesized effect modifiers.

To the best of our knowledge, no prior work has assessed whether HOMA-IR *prior to pregnancy* is associated with GDM. It is known that pregnancy increases insulin resistance to promote fetal growth, which increases the risk of conditions such as GDM; thus, it is unclear if the levels of HOMA-IR reported in these prior studies are due to changes in pregnancy or indicative of an individual's baseline insulin resistance. Our findings show that insulin resistance is present years before pregnancy, and higher degrees of biomarkers of insulin resistance are associated with the risk of GDM, suggesting that populations at higher risk for GDM could be identified prior to pregnancy or in early pregnancy.

Our findings did not reveal an association between HOMA-β and risk of GDM, suggesting that increased insulin resistance is associated with GDM but not decreased insulin secretion prior to pregnancy. This is in contrast with an existing study that shows that levels of HOMA-β in pregnancy were lower in GDM patients compared with non-GDM patients; however, this study had a smaller sample size (n = 40), had a different geographic setting (single center in Bangladesh), and did not report adjusted results [34]. Additionally, a case-control study of 1968 individuals in China reported that the highest quartile of HOMA-β was associated with increased odds of postpartum diabetes, adjusting for GDM status, among other factors (6.25; 95% CI 2.86-13.70) [13]. Prior work suggests that in the postpartum context after GDM, increasing insulin resistance can lead to increased insulin secretion to compensative until β-cell exhaustion, after which insulin secretion declines [35, 36]. So it is possible that we did not observe an association with *prepregnancy* insulin secretion because at that stage the β cells were likely still able to compensate for increasing insulin resistance.

Our findings suggest that the role of insulin resistance in contributing to GDM risk may vary by race and ethnicity. There is some evidence to support that insulin resistance could vary by race and ethnicity [14, 37]. A study assessing women with polycystic ovarian syndrome found that non-Hispanic White and Black women had higher baseline insulin resistance and β-cell response compared with Hispanic and Asian women [14]. Less is known about the association between insulin resistance or secretion and race and ethnicity with GDM. Our findings show that there are variations in the strength of the associations between HOMA-IR and GDM odds by racial and ethnic subgroups after adjusting for known GDM risk factors; specifically, we observed no significant association among non-Hispanic White or Hispanic individuals, and higher HOMA-IR was associated with elevated ORs for GDM among Asian and non-Hispanic Black individuals. This finding is somewhat unexpected because Asian individuals develop GDM at lower BMI thresholds [38]. However, it is possible that BMI does not directly correlate with HOMA-IR in young healthy populations the way it is suggested to in older, comorbid populations [39]. There is limited work examining how the association between HOMA-IR prior to pregnancy or in pregnancy and GDM is modified by race and ethnicity. One study noted that women with Asian or Arab ethnicity had higher HOMA-IR levels in pregnancy compared with women with European ethnicity, prior to a GDM diagnosis, which is consistent with our findings of a stronger association among Asian individuals [15].

Our research presents a few promising avenues for future work. Our stratified analyses are suggestive of potential effect modification of the association between HOMA-IR and GDM by race and ethnicity, although formal tests for interaction were nonsignificant. Future studies among adequately powered studies with fasted blood samples from racial and ethnic subgroups, particularly subcategories among Asians, would clarify the role of race and ethnicity. Additionally, future work could assess the predictive ability of HOMA-IR from a range of time points prior to pregnancy as an easy and relatively inexpensive way to measure biomarkers, with potential for identification of individuals at high-risk for GDM. Population stratification prior to pregnancy based on risk of GDM would allow for earlier intervention and management of hyperglycemia in pregnancy, which could reduce the burden on maternal and fetal outcomes associated with GDM.

### Strengths and Limitations

Strengths of this study include the ability to assess biomarker levels prior to pregnancy to establish clear temporality prior to GDM diagnosis, the ascertainment of GDM within our cohort using an established gestational diabetes registry, and a diverse study population that is representative of the underlying source population [23]. The findings from this study should be interpreted in context of its limitations. First, our main findings are presented in the overall sample, where we had adequate sample size; however, HOMA-IR and HOMA-β are typically measured at fasting, and only a subset of the study sample was fasting at the time of their blood collection, presented in the sensitivity analyses. Signals from the sensitivity analysis are generally in line with the overall sample; 1 notable exception was that the association for tertile 3 of HOMA-IR was significant in the sample of patients with a fasting blood draw but not the overall sample. Second, given the sample size, we are limited to examining aggregated racial and ethnic subgroups; in particular, future work should examine how the association between HOMA-IR and GDM varies by Asian subgroups, among whom the risk of GDM has been shown to vary [16]. Additionally, it is likely that our sample was underpowered for subgroups such as Hispanic individuals (n = 78), and as such, estimates among these subgroups should be interpreted with caution. Third, we did not have data on several potential confounding factors for this sample such as lifestyle factors like physical activity and diet. We also did not have direct measures of adiposity or visceral fat, which may be more associated with measures of insulin resistance than BMI. Fourth, instead of the gold standard for direct measurement of insulin resistance, the euglycemic-hyperinsulinemic clamp, we used glucose and insulin levels measured during the MHC to estimate HOMA-IR as a surrogate measure of insulin resistance, which has been shown to correlate with results from the euglycemic-hyperinsulinemic clamp [30]. Fifth, while our study is novel in its use of HOMA-IR and HOMA-β from prior to pregnancy, our interpretation of our findings includes the assumption that these measurements from a single time point, on average 7 years prior to pregnancy, represent generalized levels of insulin sensitivity and secretion over that time. Future studies should consider collecting data from multiple time points prior to pregnancy to clarify if HOMA-IR levels during specific preconception time windows predicts future risk of GDM.

In a nested case-control study of 254 GDM cases and 497 controls, we found that higher levels of HOMA-IR, assessed on average 6 years before pregnancy, were associated with elevated risk of GDM. Although our sample size was limited when stratifying by race and ethnicity, our findings suggest that the strength of association between HOMA-IR and odds of GDM could vary by race and ethnicity. We observed that higher HOMA-IR was associated with elevated odds of GDM among Asian and non-Hispanic Black women, while there was no significant association among non-Hispanic White or Hispanic women. We did not find any association between HOMA-β measured prior to pregnancy and GDM. These findings suggest that HOMA-IR from prior to pregnancy, derived from glucose and insulin that are easy and relatively inexpensive to measure, could be used to identify high-risk reproductive-aged individuals and risk stratify populations for GDM risk.

## Data Availability

The datasets analyzed for the current study are not publicly available due to Human Subjects protection provisions by the KPNC Internal Review Board, but deidentified data are available from the corresponding author upon reasonable request by contacting kpnc.irb@kp.org.
